# Learning curves for robotic pancreatic surgery-from distal pancreatectomy to pancreaticoduodenectomy

**DOI:** 10.1097/MD.0000000000013000

**Published:** 2018-11-09

**Authors:** Bor-Uei Shyr, Shih-Chin Chen, Yi-Ming Shyr, Shin-E. Wang

**Affiliations:** Department of Surgery, Taipei Veterans General Hospital, National Yang Ming University, Taipei, Taiwan.

**Keywords:** distal pancreatectomy, learning curve, pancreaticoduodenectomy, robotic

## Abstract

This study sought to identify the learning curves of console time (CT) for robotic pancreaticoduodenectomy (RPD) and robotic distal pancreatectomy (RDP). Perioperative outcomes were compared between the early group of surgeries performed early in the learning curve and the late group of surgeries performed after the learning curve.

Pancreaticoduodenectomy (PD) is a technically demanding and challenging procedure carrying a high morbidity.

Data for RDP and RPD were prospectively collected for analysis. The learning curve was assessed by cumulative sum (CUSUM). Based on CUSUM analyses, patients were divided into the early group and the late group.

There were 70 RDP and 61 RPD cases. It required 37 cases to overcome the learning curve for RDP and 20 cases for RPD. The median console time was significantly shorter in the late group for both RDP (112 minutes vs 225 minutes, *P < *.001) and RPD (360 minuntes vs 520 minutes, *P < *.001). Median blood loss was significantly less in the late group for both RDP (30 cc vs 100 cc, *P = *.003) and RPD (100 cc vs 200 cc, *P < *.001). No surgical mortality occurred in either group. Clinically relevant pancreatic fistula rate was 22.9% for RDP (32.4% in the early group vs 12.1% in the late group, P = .043), and 11.5% for RPD (0 in early group vs 17.1% in late group, *P = *.084).

This study demonstrates that the RPD learning curve is 20 cases with prior experience of RDP and confirms the safety and feasibility of both RPD and RDP. Practice and familiarity with the robotic platform are likely to contribute to significant shortening of the learning curve in robotic pancreatic surgery, while knowledge and experience, in addition to practical skills, are also essential to minimize the potential surgical risks of RPD.

## 1 Introduction

Minimally invasive surgery has become a worldwide trend in many surgical fields, including pancreatic surgery.^[1–5]^ Increasing evidence has demonstrated not only the safety and feasibility of laparoscopic pancreatectomies, but also advantages in terms of postoperative outcome and comparable oncological results.^[6–8]^ Recently, with the advantageous ergonomics of the da Vinci Robotic Surgical System (Intuitive Surgical, Inc., Sunnyvale, CA), several limitations related to the laparoscopic approach have been overcome. Moreover, given the high-quality three-dimensional and 10 to 15 magnification view, elimination of surgeon's tremor, and articulation of instruments with 540° of motion, robotic approach even allows to facilitate more complex and delicate procedures such as pancreaticoduodenectomy (PD) involving extensive dissection and the restoration of digestive tract continuity for the pancreas, bile duct, and stomach.^[3,6,9]^

PD is a technically demanding and challenging procedure carrying a high morbidity. For robotic pancreaticoduodenectomy (RPD), the learning curve would entail the mastery of some facets unique to the use of robotic surgical system, including optimal port placement, good coordination between the console surgeon and bedside assistant, and overcoming the loss of tactile feedback.^[1–3,10–14]^ Cumulative sum (CUSUM) is a mathematical inspection scheme that was first described by E.S. Page in 1954 as a method to monitor performance in the manufacturing industry, and has since been widely employed in the assessment of new technical skills.^[3,13,15–21]^ CUSUM method was adopted by the medical profession in the 1970s for the analysis of learning curves in the technical training of a variety of procedures.^[16,22,23]^ and surgical procedures.^[18,21]^ Multiple reports on robotic surgery have been published, but only a few reports have evaluated the learning curve for both robotic distal pancreatectomy (RDP)^[17,24]^ and RPD^[3,13]^.

This study was to present our experience in robotic pancreatic surgery. CUSUM analysis was conducted to identify the learning curve of the console time for RDP and RPD. Perioperative outcomes were compared between the early group of surgeries performed early in the learning curve and the late group of surgeries performed after the learning curve.

## 2 Patients and methods

Robotic distal pancreatectomy (RDP) was initiated at our institution on September 13, 2011, and the first robotic pancreaticoduodenectomy (RPD) was performed on July 15, 2014. A dedicated team, including SEW and YMS who both had performed more than 500 cases of open PD, was involved in all the robotic cases. The study was approved by our Institutional Review Board (IRB-TPEVGH No.: 2018-05-0093BC). Appropriate written informed consent was obtained from the patients. The da Vinci Si Surgical System (Intuitive Surgical, Inc., Sunnyvale, CA) was used for robotic pancreatic surgeries and all of the cases were pure robotic pancreatic surgery, with exclusion of cases needing conversion or hybrid surgery. All perioperative data were prospectively collected, including gender, age, presenting symptoms, laboratory blood data, radiologic imaging, perioperative parameters, surgical outcomes, and pathology. Operative mortality was defined as death within 30 days after operation. Postoperative pancreatic fistula (POPF) was graded according to the International Study Group for Pancreatic Fistula (ISGPF) criteria.^[25]^ Delayed gastric emptying (DEG)^[26]^ and postpancreatectomy hemorrhage (PPH)^[27]^ were identified and classified with standardized criteria defined by an international study group of pancreatic surgery. Postoperative complications were graded according to the Clavien–Dindo classification.^[28]^

For RDP, 5 ports (4 robotic trocars including a 12-mm camera port and a 12-mm accessory port) were used. A trans-umbilical incision was made for the insertion of a 12-mm camera port, and pneumoperitoneum was established at a pressure of <15 mm Hg. Three 8-mm robotic working ports were placed, with one at the midclavicular line of the left side of the patient's abdomen at the level of umbilicus. The other 2 ports were placed on the right side, with one at the anterior axillary line about 2 cm below the subcostal region and the other at the midclavicular line slightly above the umbilicus level. A 12-mm accessory port was placed in between the camera port and the left side of the robotic working port.

For RPD, 6 ports (4 robotic trocars and 2 accessory ports) were used. A trans-umbilical incision was made for the insertion of a 12-mm accessory port, and pneumoperitoneum was established at a pressure of < 15 mm Hg. A 12-mm camera port was placed. Three 8-mm robotic working ports were placed, including one at the right anterior axillary line about 2 cm below subcostal region, another one at the left midclavicular line slightly above the umbilicus level and the third at the left anterior axillary line about 2 cm below the subcostal region. The 12-mm camera port was set up on the right side about 5 cm from the umbilicus. The other 5-mm accessory port was placed on the right midclavicular line slightly below the camera port. The technique of pancreatic reconstruction in RPD was the modified Blumgart pancreaticojejunostomy (PJ), which was similar to that for our open method.^[29]^

The learning curve was assessed using the cumulative sum (CUSUM) method similar to that described by Napoli et al.^[3]^ The CUSUM was obtained by adding up the calculated difference from the overall mean, starting with the first case to the next cumulatively. This method was used for all study cases, taking into account the console time (CT). Patients were chronologically arranged from the earliest to the latest data of surgery. The CUSUM-CT for the first patient was the difference between the CT for the first patient and the mean CT for all patients. The CUSUM-CT of the second patient was the CUSUM-CT of the previous case added to the difference between the CT of the second patient and the mean CT for all patients. This same method was repeated for each patient except for the last patient, which was calculated as zero. If the CT of a case was more than the mean CT, the addition to the running value of the CUSUM-CT was a positive number (upward slope on the graph), and it was a negative number if the CT of a case was less than the mean CT (downward slope). A linear regression analysis was also performed to fit the CUSUM-CT into a model in order to detect the different phases of learning process. Based on CUSUM analyses, patients were was divided into 2 groups, early group before learning curve and late group after learning curve for both RDP and RPD.

Statistical analyses were carried out using Statistical Product and Service Solutions (SPSS) version 21.0 software (SPSS Inc., IBM, Armonk, NY). All continuous data were presented as median and mean ± standard deviation (SD), and frequencies were presented when appropriate for the type of data. Mean values of continuous variables were compared with a 2-tailed Student's *t* test. Nonparametric statistical tests were used if the variables did not follow normal distribution. Categorical variables were presented as numbers and percentages. Categorical variables were compared using Pearson's χ^2^ test or Fisher's exact test contingency tables. For all analyses, a *P* value <.05 was considered statistically significant.

## 3 Results

**Figure 1:**
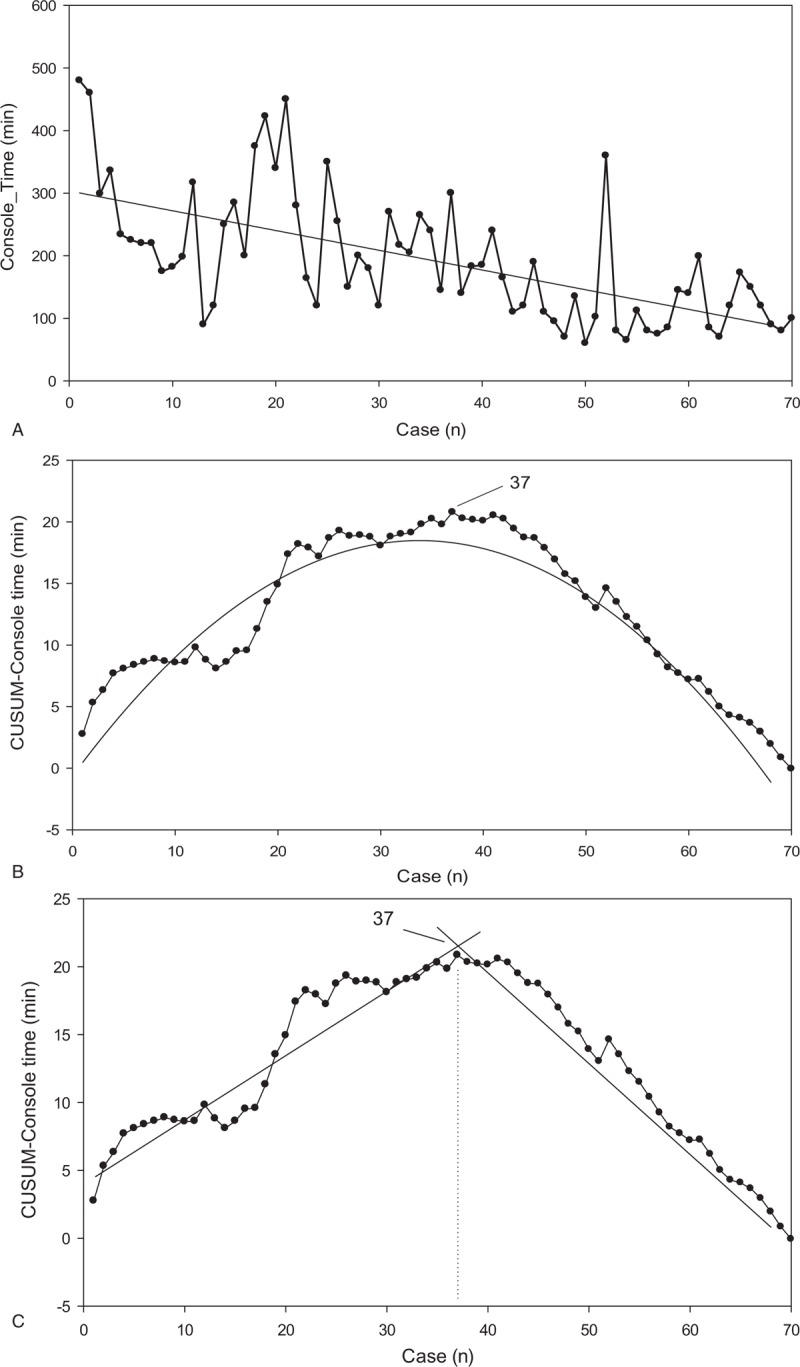
CUSUM analysis for robotic distal pancreatectomy (RDP). (A) Raw console time (CT) plotted for each RDP case arranged in chronological order. (B) Cumulative sum of the console time (CUSUM-CT) plotted against the chronological order of RDP cases modeled as a parabola. (C) Two phases of the learning process in RDP are identified using the CUSUM-CT curve by linear regression analysis. CT  = console time, CUSUM  = cumulative sum, RDP  = robotic distal pancreatectomy.

**Figure 2:**
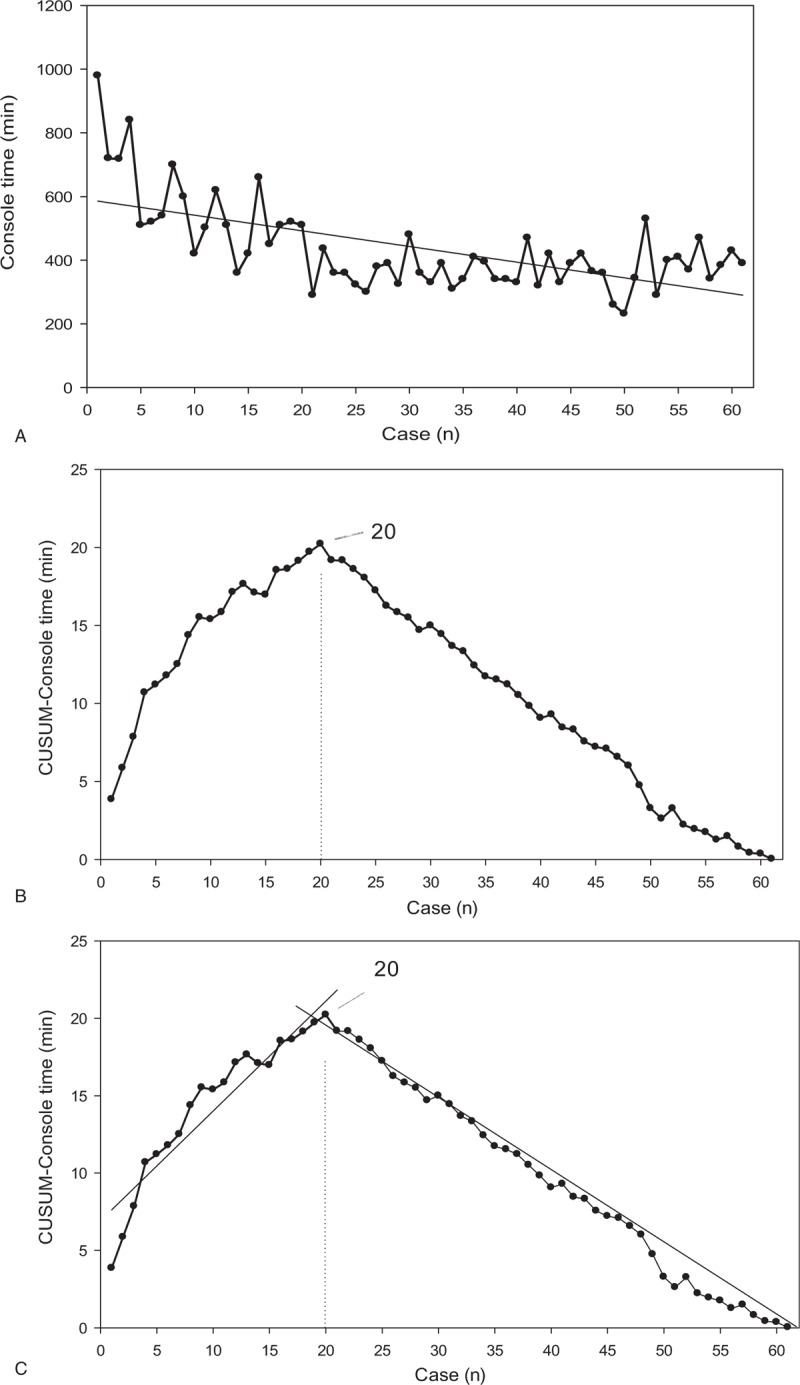
CUSUM analysis for robotic pancreaticoduodenectomy (RPD). (A) Raw console time (CT) plotted for each RPD case arranged in chronological order. (B) Cumulative sum of the console time (CUSUM-CT) plotted against the chronological order of RPD cases modeled as a parabola. (C) Two phases of the learning process in RPD are identified using the CUSUM-CT curve by linear regression analysis. CT  = console time, CUSUM  = cumulative sum, RDP  = robotic distal pancreatectomy.

There were 70 RDP and 61 RPD cases of pure robotic surgery. CUSUM-CT analysis is illustrated in Figure 1A–C for RDP and Figure 2A–C for RPD. Two distinct phases of the learning curve could be identified for both RDP and RPD. It took 37 cases to overcome the learning curve for RDP and 20 cases for RPD. Based on the identification of the learning curve of the CT, an analysis of perioperative outcomes was conducted to compare those in the learning curve cohort (early group) with those in the later procedures after the learning curve (late group). Demographics of patients undergoing RDP and RPD are shown in Table 1, and no significant differences were noted in terms of the demographic data between the early and the late groups for both RDP and RPD.

**Table 1:**
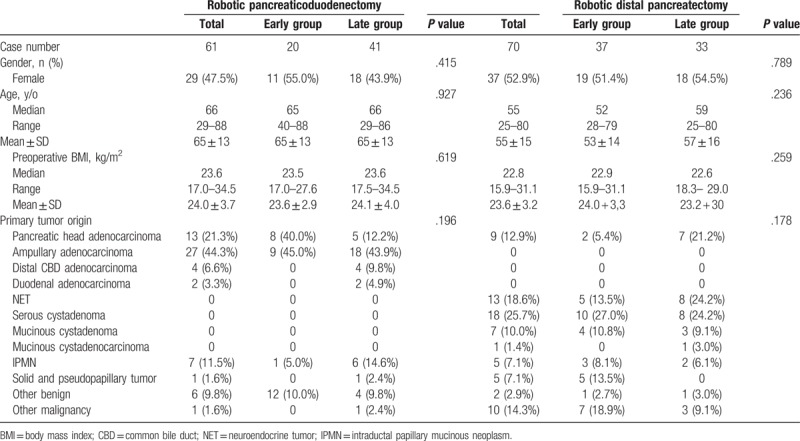
Demographics of patients undergoing robotic pancreaticoduodenectomy and distal pancreatectomy.

Table 2 lists the perioperative parameters. The median docking time was significantly shorter in the late group for both RDP (2 minutes vs 5 minutes, *P = *.008) and RPD (2 minutes vs 3 minutes, *P = *.004). The median console time was also significantly shorter in the late group for both RDP (112 minutes vs 225 minutes, *P < *.001) and RPD (360 minutes vs 520 minutes, *P < *.001). The median blood loss was significantly less in the late group for both RDP (30 cc vs 100 cc, *P = *.003) and RPD (100 cc vs 200 cc, *P < *.001).

**Table 2:**
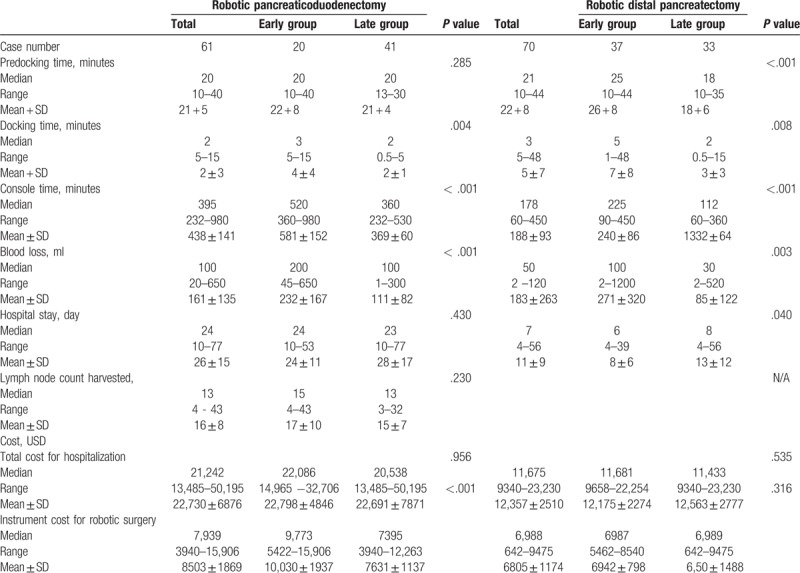
Perioperative parameters for robotic pancreaticoduodenectomy and distal pancreatectomy.

Surgical outcomes are shown in Table 3. There was no surgical mortality after RDP and RPD in this series, though the surgical morbidity rate was high at 38.6% for RDP (45.9% in the early group vs 30.3% in the late group, P = .059) and 41.0% for RPD (50.0% in the early group vs 36.6% in the late group, *P = *.708). Clinically relevant pancreatic fistula (CRPF) rate was 22.9% for RDP (32.4% in the early group vs 12.1% in the late group, P = .043) and 11.5% for RPD (0% in the early group vs 17.1% in the late group, *P = *.084). The rate of significant gastric atonia (grades B and C) was only 3.3% for RPD (5.0% in the early group vs 2.4% in the late group, *P = *.685), with no occurrence of gastric atonia for RDP. The chyle leakage rate was 8.6% for RDP (10.8% in the early group vs 6.1% in the late group, P = .479) and 8.2% for RPD (0% in the early group vs 12.2% in the late group, *P = *.162).

**Table 3:**
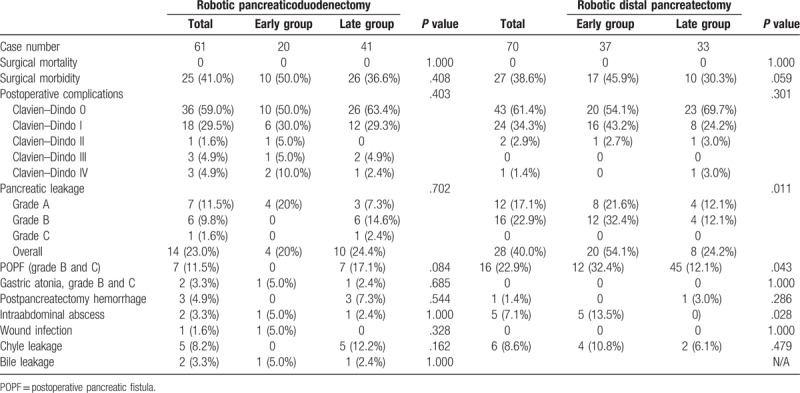
Surgical risks after robotic pancreaticoduodenectomy and distal pancreatectomy.

## 4 Discussion

PD is considered a challenging surgical procedure that involves complex surgical anatomy and extensive dissection and requires meticulous and tedious technique to restore digestive continuity. Moreover, it carries a high rate of potential postoperative morbidity such as pancreatic fistula, intraabdominal bleeding, sepsis, and delayed gastric emptying, which can contribute to a prolonged hospital stays.^[6,30,31]^ Despite its complexity, use of robotic approach for pancreatic resections has been increasing in some centers, ^[1,5,6,10]^ Perhaps the most appreciated aspect of the robotic approach is the shorter learning curve. The 7 degrees of freedom, 3-dimensional view, ergonomics, and tremor filtration are possible reasons for this reduced learning curve, as compared with the laparoscopic approach.^[32]^ Robotic approach in pancreatic surgery may be superior to the traditional open approach in terms of intraoperative blood loss, wound pain, and length of hospital stay.

In traditional open surgery, surgeons stand beside the patient, are fully embedded in the operative environment, and have the opportunity to use the full range of their surgeons’ senses, including tactile feedback. In laparoscopic surgery, surgeons are still close to the patient and embedded in the operative environment, but many of the technical advantages of open surgery are not available, such as direct tactile feedback. In the robotic surgery, surgeons operate on patients in a virtual reality system without directly contacting the patients, with a total lack of tactile feedback and without direct perception of the position of the surgical instruments outside the endoscopic vision. In light of the complete absence of haptic feedback and lack of direct visual control of the instrument position in robotic surgery, various adaptations and the establishment of a new model of team cooperation have become necessary.^[3]^ Therefore, a learning curve would exist for any surgeon embarking on performing robotic surgery irrespective of the surgeon's extent of prior experience in traditional open or laparoscopic surgery.

It has been demonstrated that surgeons with higher volumes have better outcomes, which suggests the presence of a learning curve for PD.^[33]^ In fact, the learning curve for open PD corresponds to 50 to 60 operations.^[33–35]^ In laparoscopic PD, Speicher et al^[36]^ reported that 50 cases of team-based laparoscopic PD were required for the learning curve to achieve in reducing operation time and blood loss as compared with the open approach. The reported learning curve for RPD is variable. Zureikat et al^[10]^ suggested that the learning curve resulting in comparable outcomes to the open approach in terms of surgical risks and oncological results could be overcome after 80 RPD procedures. Chen et al^[37]^ reported that comparable results could be achieved by RPD when treating pancreatic diseases after a learning curve of 40 cases. While the Pittsburgh group analyzed 200 consecutive RPD cases and showed that operation time dropped after 80 procedures,^[13]^ the Shanghai group analyzed 60 RPD cases in the context of a prospective comparison with 120 open PD cases and showed reduction in operation time, blood loss, and incidence of complications after 40 procedures.^[38–40]^

CUSUM, a cumulative summation technique, has been effectively used to assess the learning curve of a novel surgical procedure because it can display the variance from the mean on a case-by-case basis.^[17]^ In the present study, 2 distinct phases of the learning curve were identified for both RDP and RPD by CUSUM-CT analyses. It took 37 cases to complete the learning curve of RDP in the context of no prior experience in robotic surgery. Nevertheless, only 20 cases were needed for us to overcome the learning curve for RPD, which was initiated with earlier experience of performing RDP. Both the learning curves of RPD and RDP were shorter than those previously reported in the literature. With no experience in robotic surgery, but with a high volume of open PD surgeries, our team approached the integration of the robotic surgery with RDP first, and the RPD program was not initiated until nearly 3 years later. Actually, we did not have selection bias irrespective of BMI and pathological diagnosis, except significant vascular involvement by cancer. This study clearly revealed that our prior experience in RDP played a significant role in the development of skill in RPD. Practice and familiarity with the robotic platform are likely to contribute to a significantly reduced learning curve for a complex and risky procedure such as RPD

Minimization of blood loss has been one of the benefits provided by the robotic approach, which was reported to range from 100 to 634 mL for RPD.^[6,13]^ In this study, we also showed that the medians of blood loss were significantly less in the late groups compared to the early group (100 mL vs 200 mL for RPD and 100 mL vs 30 mL for RDP, respectively), which could be a reflection of improved tissue dissection using the robotic approach after the learning curve. However, other major surgical parameters including harvested lymph node count, surgical morbidity, and pancreatic leakage were not significantly different before and after the learning curve for RPD. In this study, we had no surgical mortality after robotic pancreatic surgeries. Our results suggest that surgical outcomes may not differ when the robotic approach is used for PD by high-volume surgeons who possess sound expertise in open PD. In other words, surgical knowledge and experience, in addition to practical skills, are also essential to minimize surgical risks in attempting robotic pancreatic surgery.

In conclusion, this study has identified a RPD learning curve of 20 cases in the context of prior experience of RDP, and has confirmed the safety and feasibility of both RPD and RDP. Practice and familiarity with the robotic platform are likely to contribute to the significant shortening of the learning curve in robotic pancreatic surgery. A shorter learning curve for RPD can be predicted based on robust prior experience in open and robotic surgery. Therefore, surgical knowledge and experience, in addition to practical skills, are essential to minimize the potential surgical risks of robotic pancreatic surgery.

## Author contributions

**Conceptualization:** Yi-Ming Shyr, Shin-E Wang.

**Data curation:** Bor-Uei Shyr, Shih-Chin Chen, Yi-Ming Shyr, Shin-E Wang.

**Formal analysis:** Bor-Uei Shyr, Shih-Chin Chen, Yi-Ming Shyr, Shin-E Wang.

**Funding acquisition:** Yi-Ming Shyr, Shin-E Wang.

**Investigation:** Bor-Uei Shyr, Shih-Chin Chen, Yi-Ming Shyr, Shin-E Wang.

**Methodology:** Bor-Uei Shyr, Shih-Chin Chen, Yi-Ming Shyr, Shin-E Wang.

**Project administration:** Shin-E Wang.

**Resources:** Bor-Uei Shyr, Shih-Chin Chen, Yi-Ming Shyr, Shin-E Wang.

**Software:** Shin-E Wang.

**Supervision:** Bor-Uei Shyr, Yi-Ming Shyr, Shin-E Wang.

**Validation:** Bor-Uei Shyr, Yi-Ming Shyr, Shin-E Wang.

**Visualization:** Yi-Ming Shyr, Shin-E Wang.

**Writing – original draft:** Bor-Uei Shyr, Yi-Ming Shyr, Shin-E Wang.

**Writing – review & editing:** Bor-Uei Shyr, Yi-Ming Shyr, Shin-E Wang.

## References

[1] Boggi U Signori S De Lio N Feasibility of robotic pancreaticoduodenectomy. Br J Surg 2013;100:917–25. 23640668 10.1002/bjs.9135

[2] Napoli N Kauffmann EF Menonna F Indications, technique, and results of robotic pancreatoduodenectomy. Updates Surg 2016;68:295–305. 27614901 10.1007/s13304-016-0387-7

[3] Napoli N Kauffmann EF Palmeri M The learning curve in robotic pancreaticoduodenectomy. Dig Surg 2016;33:299–307. 27215422 10.1159/000445015

[4] Stafford AT Walsh RM Robotic surgery of the pancreas: the current state of the art. J Surg Oncol 2015;112:289–94. 26220683 10.1002/jso.23952

[5] Zureikat AH Moser AJ Boone BA 250 robotic pancreatic resections: safety and feasibility. Ann Surg 2013;258:559–62. 554-9; discussion. 10.1097/SLA.0b013e3182a4e87c PMC4619895 24002300

[6] Memeo R Sangiuolo F de Blasi V Robotic pancreaticoduodenectomy and distal pancreatectomy: State of the art. J Visc Surg 2016;153:353–9. 27185566 10.1016/j.jviscsurg.2016.04.001

[7] Palanivelu C Rajan PS Rangarajan M Evolution in techniques of laparoscopic pancreaticoduodenectomy: a decade long experience from a tertiary center. J Hepatobiliary Pancreat Surg 2009;16:731–40. 19652900 10.1007/s00534-009-0157-8

[8] Song KB Kim SC Hwang DW Matched case-control analysis comparing laparoscopic and open pylorus-preserving pancreaticoduodenectomy in patients with periampullary tumors. Ann Surg 2015;262:146–55. 25563866 10.1097/SLA.0000000000001079

[9] Liu R Zhang T Zhao ZM The surgical outcomes of robot-assisted laparoscopic pancreaticoduodenectomy versus laparoscopic pancreaticoduodenectomy for periampullary neoplasms: a comparative study of a single center. Surg Endosc 2017;31:2380–466. 27631318 10.1007/s00464-016-5238-6

[10] Zureikat AH Postlewait LM Liu Y A multi-institutional comparison of perioperative outcomes of robotic and open pancreaticoduodenectomy. Ann Surg 2016;264:640–9. 27433907 10.1097/SLA.0000000000001869

[11] Baker EH Ross SW Seshadri R Robotic pancreaticoduodenectomy for pancreatic adenocarcinoma: role in 2014 and beyond. J Gastrointest Oncol 2015;6:396–405. 26261726 10.3978/j.issn.2078-6891.2015.027 PMC4502160

[12] Baker EH Ross SW Seshadri R Robotic pancreaticoduodenectomy: comparison of complications and cost to the open approach. Int J Med Robot 2016;12:554–60. 26202591 10.1002/rcs.1688

[13] Boone BA Zenati M Hogg ME Assessment of quality outcomes for robotic pancreaticoduodenectomy: identification of the learning curve. JAMA Surg 2015;150:416–22. 25761143 10.1001/jamasurg.2015.17

[14] Cirocchi R Partelli S Trastulli S A systematic review on robotic pancreaticoduodenectomy. Surg Oncol 2013;22:238–46. 24060451 10.1016/j.suronc.2013.08.003

[15] Hu Y Jolissaint JS Ramirez A Cumulative sum: a proficiency metric for basic endoscopic training. J Surg Res 2014;192:62–7. 24976441 10.1016/j.jss.2014.05.056 PMC4188705

[16] Naik VN Devito I Halpern SH Cusum analysis is a useful tool to assess resident proficiency at insertion of labour epidurals. Can J Anaesth 2003;50:694–8. 12944444 10.1007/BF03018712

[17] Shakir M Boone BA Polanco PM The learning curve for robotic distal pancreatectomy: an analysis of outcomes of the first 100 consecutive cases at a high-volume pancreatic centre. HPB (Oxford) 2015;17:580–6. 25906690 10.1111/hpb.12412 PMC4474504

[18] Wohl H The cusum plot: its utility in the analysis of clinical data. N Engl J Med 1977;296:1044–5. 846547 10.1056/NEJM197705052961806

[19] Melich G Hong YK Kim J Simultaneous development of laparoscopy and robotics provides acceptable perioperative outcomes and shows robotics to have a faster learning curve and to be overall faster in rectal cancer surgery: analysis of novice MIS surgeon learning curves. Surg Endosc 2015;29:558–68. 25030474 10.1007/s00464-014-3698-0

[20] Park EJ Kim CW Cho MS Multidimensional analyses of the learning curve of robotic low anterior resection for rectal cancer: 3-phase learning process comparison. Surg Endosc 2014;28:2821–31. 24902812 10.1007/s00464-014-3569-8

[21] Chaput de Saintonge DM Vere DW Why don’t doctors use cusums? Lancet 1974;1:120–1. 4130314 10.1016/s0140-6736(74)92345-9

[22] Hu Y Puri V Crabtree TD Attaining proficiency with endobronchial ultrasound-guided transbronchial needle aspiration. J Thorac Cardiovasc Surg 2013;146:1387–92. 24075565 10.1016/j.jtcvs.2013.07.077 PMC3981557

[23] East JM Valentine CS Kanchev E Sentinel lymph node biopsy for breast cancer using methylene blue dye manifests a short learning curve among experienced surgeons: a prospective tabular cumulative sum (CUSUM) analysis. BMC Surg 2009;9:2. 19173714 10.1186/1471-2482-9-2 PMC2640353

[24] Napoli N Kauffmann EF Perrone VG The learning curve in robotic distal pancreatectomy. Updates Surg 2015;67:257–64. 25990666 10.1007/s13304-015-0299-y

[25] Bassi C Marchegiani G Dervenis C The 2016 update of the International Study Group (ISGPS) definition and grading of postoperative pancreatic fistula: 11 Years After. Surgery 2017;161:584–91. 28040257 10.1016/j.surg.2016.11.014

[26] Wente MN Bassi C Dervenis C Delayed gastric emptying (DGE) after pancreatic surgery: a suggested definition by the International Study Group of Pancreatic Surgery (ISGPS). Surgery 2007;142:761–8. 17981197 10.1016/j.surg.2007.05.005

[27] Wente MN Veit JA Bassi C Postpancreatectomy hemorrhage (PPH): an International Study Group of Pancreatic Surgery (ISGPS) definition. Surgery 2007;142:20–5. 17629996 10.1016/j.surg.2007.02.001

[28] Dindo D Demartines N Clavien P-A Classification of surgical complications. Annals of Surgery 2004;240:205–13. 15273542 10.1097/01.sla.0000133083.54934.ae PMC1360123

[29] Wang SE Chen SC Shyr BU Comparison of modified Blumgart pancreaticojejunostomy and pancreaticogastrostomy after pancreaticoduodenectomy. HPB (Oxford) 2016;18:229–35. 27017162 10.1016/j.hpb.2015.09.007 PMC4814607

[30] Memeo R Tzedakis S de Blasi V Robotic pancreaticoduodenectomy: operative steps (with video). Surg Laparosc Endosc Percutan Tech 2016;26:e91–4. 27636149 10.1097/SLE.0000000000000304

[31] Joyce D Morris-Stiff G Falk GA Robotic surgery of the pancreas. World J Gastroenterol 2014;20:14726–32. 25356035 10.3748/wjg.v20.i40.14726 PMC4209538

[32] Lebeau T Roupret M Ferhi K The role of a well-trained team on the early learning curve of robot-assisted laparoscopic procedures: the example of radical prostatectomy. Int J Med Robot 2012;8:67–72. 22556136 10.1002/rcs.435

[33] Fisher WE Hodges SE Wu MF Assessment of the learning curve for pancreaticoduodenectomy. Am J Surg 2012;203:684–90. 22079032 10.1016/j.amjsurg.2011.05.006

[34] Schmidt CM Turrini O Parikh P Effect of hospital volume, surgeon experience, and surgeon volume on patient outcomes after pancreaticoduodenectomy: a single-institution experience. Arch Surg 2010;145:634–40. 20644125 10.1001/archsurg.2010.118

[35] Tseng JF Pisters PW Lee JE The learning curve in pancreatic surgery. Surgery 2007;141:694–701. 17511115 10.1016/j.surg.2007.04.001

[36] Speicher PJ Nussbaum DP White RR Defining the learning curve for team-based laparoscopic pancreaticoduodenectomy. Ann Surg Oncol 2014;21:4014–9. 24923222 10.1245/s10434-014-3839-7

[37] Chen S Chen JZ Zhan Q Robot-assisted laparoscopic versus open pancreaticoduodenectomy: a prospective, matched, mid-term follow-up study. Surg Endosc 2015;29:3698–711. 25761559 10.1007/s00464-015-4140-y

[38] Kim SC Song KB Jung YS Short-term clinical outcomes for 100 consecutive cases of laparoscopic pylorus-preserving pancreatoduodenectomy: improvement with surgical experience. Surg Endosc 2013;27:95–103. 22752284 10.1007/s00464-012-2427-9

[39] Asbun HJ Stauffer JA Laparoscopic vs open pancreaticoduodenectomy: overall outcomes and severity of complications using the Accordion Severity Grading System. J Am Coll Surg 2012;215:810–9. 22999327 10.1016/j.jamcollsurg.2012.08.006

[40] Croome KP Farnell MB Que FG Total laparoscopic pancreaticoduodenectomy for pancreatic ductal adenocarcinoma: oncologic advantages over open approaches? Ann Surg 2014;260:633–8. discussion 638-40. 25203880 10.1097/SLA.0000000000000937

